# Correlation and Similarity between Cerebral and Non-Cerebral Electrical Activity for User’s States Assessment

**DOI:** 10.3390/s19030704

**Published:** 2019-02-09

**Authors:** Gianluca Borghini, Pietro Aricò, Gianluca Di Flumeri, Nicolina Sciaraffa, Fabio Babiloni

**Affiliations:** 1 Department of Molecular Medicine, Sapienza University of Rome, Piazzale Aldo Moro, 5, 00185 Rome, Italy; pietro.arico@uniroma1.it (P.A.); gianluca.diflumeri@uniroma1.it (G.D.F.); fabio.babiloni@uniroma1.it (F.B.); 2 BrainSigns srl, via Sesto Celere, 00152 Rome, Italy; nicolina.sciaraffa@brainsigns.com; 3 IRCCS Fondazione Santa Lucia, Neuroelectrical Imaging and BCI Lab, Via Ardeatina, 306, 00179 Rome, Italy; 4 Department Anatomical, Histological, Forensic & Orthopedic Sciences, Sapienza University of Rome, Piazzale Aldo Moro, 5, 00185 Rome, Italy; 5 College of Computer Science and Technology, Hangzhou Dianzi University, Hangzhou 310018, China

**Keywords:** brain activity, EEG, human tissues conductibility, electrical activity, human body, machine-learning analysis, individual alpha frequency, correlation, biopotentials, mental states assessment

## Abstract

Human tissues own conductive properties, and the electrical activity produced by human organs can propagate throughout the body due to neuro transmitters and electrolytes. Therefore, it might be reasonable to hypothesize correlations and similarities between electrical activities among different parts of the body. Since no works have been found in this direction, the proposed study aimed at overcoming this lack of evidence and seeking analogies between the brain activity and the electrical activity of non-cerebral locations, such as the neck and wrists, to determine if i) cerebral parameters can be estimated from non-cerebral sites, and if ii) non-cerebral sensors can replace cerebral sensors for the evaluation of the users under specific experimental conditions, such as eyes open or closed. In fact, the use of cerebral sensors requires high-qualified personnel, and reliable recording systems, which are still expensive. Therefore, the possibility to use cheaper and easy-to-use equipment to estimate cerebral parameters will allow making some brain-based applications less invasive and expensive, and easier to employ. The results demonstrated the occurrence of significant correlations and analogies between cerebral and non-cerebral electrical activity. Furthermore, the same discrimination and classification accuracy were found in using the cerebral or non-cerebral sites for the user’s status assessment.

## 1. Introduction

The human body is beautifully complex, consisting of mechanical, electrical, and chemical systems that allow us to live and function. An example of a mechanical system in the body is the actin and myosin filaments found in muscles that allow them to contract. Chemical systems include the neuro transmitters that are released by neurons for communication with other cells. Finally, electrical systems include the electrical potentials that propagate down nerve cells and muscle fibers [1]. Nerve and muscle cells in the body communicate with each other via action potentials. Action potentials are voltage impulses that propagate along a nerve or muscle and may cause neurotransmitter release when the action potential reaches a specific area of the nerve cell [2]. These voltage impulses arise from tiny currents in the nerve or muscle cells. These currents are a result of charged ions flowing in and out of voltage-gated channels in the membrane of the cells. Kirchoff’s Law from basic circuits tells us that *V = IR*, where *V* is a measured voltage, *I* is a current, and *R* is resistance. In general, electrical admittivity of human tissues may be predominantly conductive or capacitive, or a combination of these, depending on tissue type, availability of charge carriers, and frequency of the applied electric field, and it is carried out by ions as well as holes [3]. Tissue homogeneity is important regarding current paths in human tissues, and from an electrical point of view, the tissue is as good as inhomogeneous. Such anisotropy is present all the way from lipids and cell membranes at a micro level, up to organs and long muscle fibres. Electrolytes, both intra and extracellular, are responsible for the conductivity of the order of 1 (S/m) and are considered to be more or less frequency independent up to roughly 10 (MHz) [4]. The dominant factor regarding charge carriers in biological materials are ions, but also purely electronic currents have been reported to take place, for instance in DNA molecules [3]. Since the cell membrane has a specific resistance, the ionic currents flowing across the membrane of the cell create a voltage, i.e., a Biopotential. These potentials are responsible for brain functions, muscle movements, cardiac functions, eye movements, sensory functions and many other events in the body. One of the most popular electrophysiological measurements is the Electrocardiogram (ECG). As the heart pumps blood, different chambers of the cardiac muscle are activated in a specific order. The ECG waveform represents the rhythmical depolarization and repolarization of each chamber of the heart. Another source of human biopotentials is the Electroencephalogram (EEG), which measures the electrical activity of neurons in the brain. Such neurophysiological signals are usually gathered by sensors placed on the skin, in particular on the chests or on the scalp, respectively. Electrical potentials measured on the skin are not the result of a single cell but are the combination of a large number of cells firing together [2]. These cells may be firing synchronously or asynchronously. In this regard, it is well known how most of the brain neurons fire synchronously when the eyes are closed, and this phenomenon can be assessed by estimating the signal frequency power (i.e., the Power Spectral Density, PSD) of the brain electrical activity (i.e., EEG signal) within the Alpha band, and see the prominence of the Alpha peak with respect to the rest of the spectra [5,6,7]. Then, when the eyes open, brain neurons start firing at different frequencies (i.e., asynchronously), since the surrounding information is consciously and unconsciously processed [8,9,10,11]. The Alpha peak will decrease significantly, whereas the other EEG rhythms (e.g., Theta band) increase. In addition, during the last decade, several studies have addressed the relationship between changes in EEG parameters after opening or closing the eyes, and metabolic responses [12,13]. One widely accepted distinction between the two conditions is that eyes-closed could define a more resting state as compared with eyes-open [14]. Accordingly, the eyes-closed state induces functional inhibition of the brain, and the eyes-open is a state for functional activation. Based on these two properties, conductibility of human tissues and synchronicity of brain neurons while keeping the eyes closed, the proposed study aimed at i) investigating the similarities between the brain (*EEG*) and body (*noEEG*) electrical activity in terms of cerebral parameters estimation, for example, the Individual Alpha Frequency (IAF) [15], and ii) measuring the capability of such *noEEG* locations to be used for the assessment of the participants’ state, for example, classifying the closed or open eyes state with respect to the EEG locations in the Delta, Theta, Alpha and Beta EEG bands. In fact, most of the commercial EEG sensors and devices, not designed for research or clinical purposes, are not capable of providing signals able to discriminate when the user has their eyes opened or closed [16]. Furthermore, the capability of using non-cerebral sensors would have important impacts on brain-based applications in real settings [17,18,19]. In fact, cerebral sensors require high-qualified personnel, and reliable recording systems are still quite expensive. Therefore, cheaper and easy-to-use equipment to estimate cerebral parameters will allow making brain-based user’s assessment less invasive and expensive, and easier to employ in everyday life. For example, non-cerebral sensors would be used to measure the user’s mental workload [20], cognitive training level [21], or pleasantness [22] while dealing with particular tasks or activities, by placing few disposable electrodes around the user’s neck. In this regard, sensors were placed over the brain scalp, around the neck, and wrist of 12 participants and their *EEG* and *noEEG* electrical activities were recorded while keeping eyes open (OA) or closed (OC), and then analyzed to find out the capability of non-cerebral electrical activity in discriminating when the participants had the eyes open or closed. 

## 2. Materials and Methods

### 2.1. Participants

Informed consent for both study participation and publication of images was obtained from a group of 12 Students (27.6 ± 3.7 years old) from the Sapienza University of Rome (Italy) after the explanation of the study. The participants were selected in order to have homogeneous experimental groups in terms of age and psychophysical conditions. The experiment was conducted following the principles outlined in the Declaration of Helsinki of 1975, as revised in 2000. The study protocol received a favorable opinion from and was approved by the Ethical Committee of the Sapienza University of Rome. The study involved only healthy participants, recruited on a voluntary basis. Only aggregate information was released, while no individual information was or will be diffused in any form.

### 2.2. Experimental Protocol

**Figure 1:**
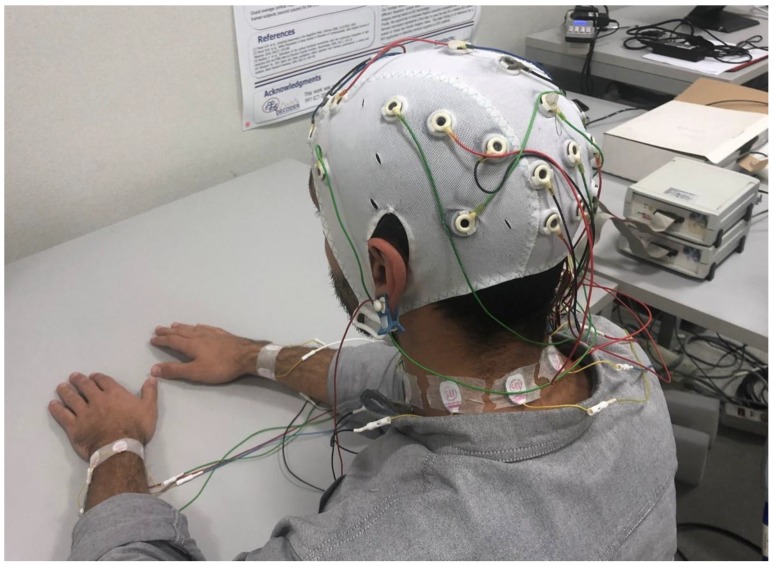
Participant wearing the electroencephalogram *(EEG)* cap and the *noEEG* sensors around the neck and wrists while seating comfortably and keeping the eyes closed.

The participants were asked to sit comfortably and relaxed in front of a blank wall. Then, they were asked to keep eyes closed (OC) or open (OA) in a random sequence of 8 repetitions of 2 min each under the instructions of the technician. The electrical activity coming from the brain (*EEG* sites), neck and wrists (*noEEG* sites) were collected along the entire experimental session. Figure 1 shows a participant during the OC condition wearing the *EEG* and *noEEG* sensors. 

### 2.3. Brain and Body Electrical Activity Recording and Analysis

**Figure 2:**
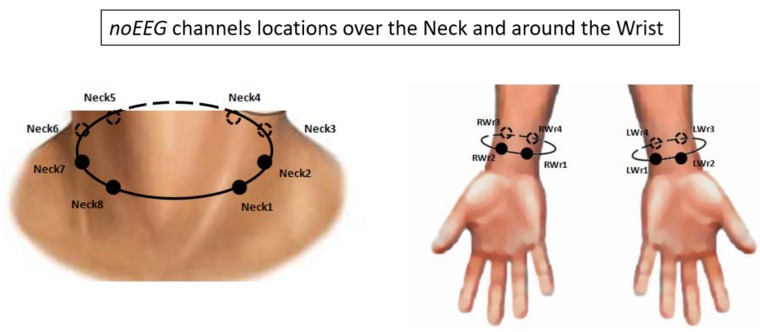
Schematic representation of the non-cerebral (*noEEG*) channels locations and labels over the neck and wrists.

The neurophysiological signals were recorded using the digital monitoring *BrainAmp* system (Brain Products GmbH, Gilching, Germany). The 27-EEG channels (*Fp1*, *Fp2*, *AF3*, *AFz*, *AF4*, *F7*, *F3, Fz*, *F4*, *F8*, *C3*, *Cz*, *C4*, *CP3*, *CPz*, *CP4*, *P7*, *P3*, *Pz*, *P4*, *P8*, *PO3*, *POz*, *PO4*, *O1*, *Oz*, *O2*), and 16-noEEG channels (*Neck1* ÷ *Neck8*, *LWrist1* ÷ *4*, *RWrist1* ÷ *4*) were collected with a sampling frequency of 250 Hz. All the electrodes were referenced to both the earlobes, grounded to *Fpz*, and the impedances of the electrodes were kept below 10 kΩ. Figure 2 shows the locations and labels of the *noEEG* channels. The acquired signals were digitally band-pass filtered by an 8th order Butterworth filter (low-pass filter cut-off frequency: 20 Hz, high-pass filter cut-off frequency: 1 Hz. 

Independent Component Analysis (ICA) was performed using all the EEG and noEEG channels to remove contributions due to muscular and heart activity. Specifically, the ICs corresponding to the Electromyogram (EMG) and the Electrocardiogram (ECG) contributions were removed from the dataset for each participant. Then, eyeblink artifacts were removed by using the *Reblinca* method [23]. The *Reblinca* allows us to correct the dataset from eyeblink artifacts without requiring the Electrooculogram (EOG) channel but simply using the *Fpz* EEG channel. Also, with respect to other regressive algorithms (e.g., Gratton method [24]) the *Reblinca* presents the advantages of preserving EEG information in blink-free signal segments by using a specific threshold criterion that automatically recognizes the occurrence of an eyeblink, and only in this case, the method corrects the dataset. If there is not any blink, the method will not introduce any changes to the signal. For other sources of artifacts, specific procedures of the EEGLAB toolbox were applied [25]. In particular, two methods were used: The threshold criterion, and the trend estimation. In the threshold criterion, an epoch was marked as “artifact” if the signal amplitude was higher than ±100 (μV), while in the trend estimation the epoch was interpolated in order to check the slope of the trend within the considered epoch. If such slope was higher than 10 (µV/s), the considered epoch would be marked as “artifact”. At the end, the epochs marked as “artifact” were removed to obtain an *artifact-free* dataset which was used for further analysis. The dataset was then segmented in 4-s epochs, overlapped of 3.9 (s), with the aim to have both a high number of observations in comparison with the number of variables and respect the condition of stationarity of the EEG signal [26,27]. In fact, the EEG signal is non-stationary, and its statistical characteristics will likely change with time. The basic source of the observed non-stationarity EEG signal is not due to the casual influences of the external stimuli on the brain mechanisms, but rather it is a reflection of switching of the inherent metastable states of neural assemblies during brain functioning [28]. However, it has been demonstrated that the EEG signal can be retained quasi-stationary within epochs of 4 s [26]. The Power Spectral Density (PSD) was then estimated by using the Fast Fourier Transform (FFT) on the artifact-free dataset with 4 s Hanning windows, overlapped of 3.9 s. Therefore, a frequency resolution of 0.25 Hz was achieved. The mean *OC* and mean *OA* conditions were calculated by averaging the PSDs of the corresponding *OC_i_* and *OA_i_* repetitions, respectively, where *i* = 1, 2, 3, 4. The Individual Alpha Frequency (IAF) was then calculated as described in Klimesch et al. [15] to define the EEG frequency bands (i.e., Delta, Theta, Alpha, and Beta) for each participant. In particular, the Alpha frequency is defined in terms of peak or gravity frequency within the traditional Alpha frequency range (f1 ÷ f2) of about 7.5–12.5 Hz. In other words, the Alpha peak frequency is that spectral component within f1 ÷ f2, which shows the largest power estimate, or it can also be calculated in terms of gravity or ‘mean’ frequency, which is the weighted sum of the spectral estimates, divided by the alpha power, in the range of f1 ÷ f2. Particularly, if there are multiple peaks in the alpha range for classification [29], gravity frequency appears a more adequate estimate of alpha frequency [15]. Once derived the Alpha peak (i.e., IAF value) was used to define the remaining EEG bands as reported in Table 1. 

### 2.4. Correlation Analysis and IAF Comparison

The first aim of the study was to demonstrate the existence of analogies between non-cerebral and cerebral locations in terms of electrical activity content. In particular, we investigated this aspect by initially exploring the correlations between the brain activity and the electrical activity recorded from the neck and wrists sites (Figure 2), and then comparing the estimations of the IAF parameter among the cerebral (*EEG*) and non-cerebral (*noEEG*) locations, reporting positive and significant correlations. In this regard, Pearson’s correlations between *EEG* and *noEEG* channels were performed on each frequency bin of the Alpha band during the mean OC condition. Only *EEG*-*noEEG* couples exhibiting positive (*R* > 0) and significant (*p* < 0.05) correlations among all the participants were considered in order to identify the common patterns. In other words, if just a participant reported non-positive or non-significant correlation, the corresponding *EEG*-*noEEG* couple would not be considered in the common pattern. Thereafter, for each participant, the IAF values were estimated in the corresponding *EEG*-*noEEG* channels of the common pattern, respectively called IAF_EEG_ and IAF_noEEG_, and then the differences were (IAF_EEG_-IAF_noEEG_) calculated. Finally, such differences were averaged across the participants to quantify the accuracy, in terms of Hz, in estimating the IAF parameter. In particular, when the averaged value was “zero”, it meant there was no difference in estimating the IAF using *EEG* or *noEEG* channels.

### 2.5. OA versus OC Discrimination Analysis 

**Figure 3:**
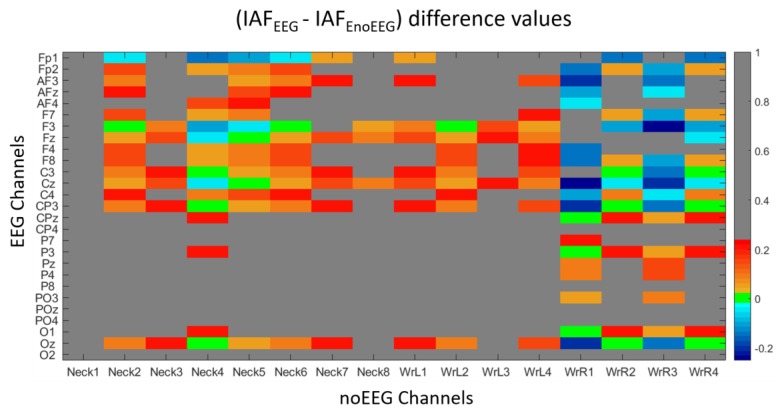
Values coming from the differences between cerebral (IAF_EEG_) and non-cerebral (IAF_noEEG_) IAF estimations. Red colors indicate the IAF_EEG_ estimations were higher than the IAF_noEEG_ estimations, while blue colors when the IAF_EEG_ estimations were lower than the IAF_noEEG_ estimations. On the contrary, green colors mean no difference existed, and differences higher than 0.25 were colored in grey.

In order to assess the discrimination and classification accuracy of the *EEG* and *noEEG* channels, a two-class Stepwise Linear Discriminant Analysis (SWLDA) classifier was used to select the most relevant spectral features among those *EEG-noEEG* channels, which exhibited the same IAF estimation (Figure 3) to discriminate the experimental conditions (i.e., OC and OA). The SWLDA consists of the combination of the forward and the backward stepwise analyses, where the input features are weighted by using ordinary least-squares regression to predict the target class labels. The method starts by creating an initial model of the discriminant function in which the most statistically significant feature is added to the model for predicting the target labels (pval*_nm_* < αENTER), where pval*_nm_* represents the p-value of the *m*-th feature at the *n*-th iteration. Then, at each new iteration, a new term was added to the model (if pval*_nm_* < αENTER). If there were not more features that satisfied this condition, a backward elimination analysis will be performed to remove the least statistically significant feature (if pval*_nm_* > αREMOVE) from the model. This process goes on unless there are no more features satisfying the entry (αENTER) and the removal (αREMOVE) conditions [30]. The standard SWLDA algorithm uses αENTER = 0.05 and αREMOVE = 0.1. Each couple of the OA*_i_* and OC*_i_* repetitions (*i* = 1, 2, 3, 4) was used as a calibration dataset, whereas the three remaining ones were used as a testing dataset. For example, with the couple OC_1_ and OA_1_, the first repetition of OC and OA was used to calibrate the SWLDA, and then it was tested on the three remaining *i*-th repetitions (*i* = 2, 3, 4). Then, the couple OC_2_ and OA_2_, the second repetition of the OC and OA, was used to calibrate the SWLDA, and then it was tested on the three remaining *i*-th repetitions (*i* = 1, 3, 4). The process went on for all the possible cross-validation combinations. In order to obtain a frequency resolution of 0.25 Hz in terms of signal power spectrum, the signals were segmented into epochs of 4 s, overlapped of 3.9 s, and since the duration of each OC and OA repetition was of 2 min, thus 120 s, the number of trials for each repetition was of (120 − 4)/0.1 = 1160. Considering that the calibration and testing dataset consisted in the OC-OA conditions, the total number of trials of the calibration and testing dataset would ideally be 1160 + 1160 = 2320. After the artifact’s rejection step, the average number of trials for the calibration and testing of the SWLDA in each cross-validation was about of 2274 ± 38. In this regard, the Linear Discriminant Function was calculated for each testing dataset, and then the average Area Under Curve (AUC) of Receiver Operating Characteristic (ROC) [31] and Classification Accuracy (ACC) [32] values were calculated considering all the possible cross-validation among the repetitions to evaluate the performance of the classifier when using only *EEG* (AUC_EEG_, and ACC_EEG_, respectively) or only *noEEG* (AUC_noEEG_, and ACC_noEEG_, respectively) features. The AUC of a classifier is equivalent to the probability that the classifier will rank a randomly chosen positive instance higher than a randomly chosen negative instance. This is equivalent to the Wilcoxon test of ranks [33]. Therefore, an AUC = 0.5 for a means random classification. On the contrary, AUC > 0.7 means good discrimination [34]. Finally, two-tail paired *t*-tests (α = 0.05) were performed on the averaged AUC_EEG_-AUC_noEEG_, and ACC_EEG_ - ACC_noEEG_ in the considered EEG bands (i.e., Delta, Theta, Alpha, and Beta) to find out i) the capability of the electrical activity coming from the *noEEG* channels to discriminate and classify the participants status, and ii) eventual differences with respect to using the EEG channels. Concerning the training and testing features, they were selected among the channels, which exhibited the same IAF estimations (Figure 3). In particular, the *EEG* features were selected among the *F3*, *Fz*, *C3*, *Cz*, *CP3*, *CPz*, *P3*, *O1*, and *Oz* channels, whereas the *noEEG* feature were selected among the *Neck2*, *Neck4*, *Neck5*, *Neck6* (neck channels), *WrL2* (left wrist channels), *WrR1*, *WrR2*, *WrR4* (right wrist channels) within the considered frequency bands (i.e., Delta, Theta, Alpha, and Beta). Table 2 reports detailed information regarding the number of features for each frequency band and channel type calculated as (#channels * #frequency bins).

## 3. Results

### 3.1. Correlation and IAF Estimation 

Figure 3 shows the values coming from the difference (IAF_EEG_-IAF_noEEG_), that is between the IAF estimations derived by EEG (IAF_EEG_) and noEEG (IAF_noEEG_) channels within EEG-noEEG couples reporting positive (R > 0) and significant (*p* < 0.05) correlation. In particular, red colors indicate when IAF_EEG_ estimations were higher than the IAF_noEEG_ estimations, while blue colors point out when the IAF_EEG_ estimations were lower than the IAF_noEEG_ estimations. On the contrary, green colors mean no difference existed, that is (IAF_EEG_-IAF_noEEG_) = 0, therefore estimating the IAF by using the considered EEG or noEEG channel was exactly the same. Differences higher than 0.25 (Hz), that is (IAF_EEG_-IAF_noEEG_) > 0.25, were colored in grey.

**Figure 4:**
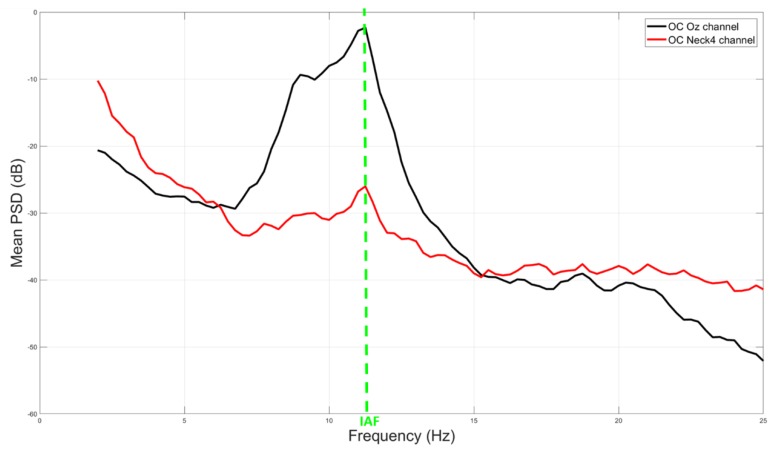
Representative example of averaged power spectra amplitude difference between *EEG* (black line) and *noEEG* (red line) channels. The green arrows highlight the consistence of the Alpha peaks (i.e., IAF value) of the brain (Oz channel) and body (Neck4 channel) spectra in the mean OC condition.

Figure 4 shows an example of averaged power spectra amplitude between a representative EEG channel (Oz, black color) and noEEG channel (Neck4, red color) of a subject. Such channels have been selected among those reporting exact estimation of the IAF (Figure 3). The green vertical line highlights the consistence of the Alpha peaks (i.e., IAF value) of the cerebral (black line) and non-cerebral (red line) spectra in the mean OC condition. Specifically, the IAF value was of 11 Hz. PSDs were normalized in dB by the formula *PSD_db_ = 20 × log10 (PSD)*.

### 3.2. OA and OC Discrimination

**Figure 5:**
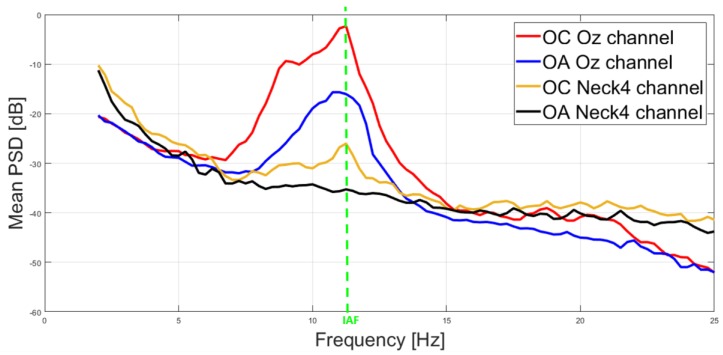
Similitude of the averaged spectra estimated from EEG (Oz) and noEEG (Neck4) channel. In particular, the red and blue lines show the averaged spectra of the EEG channel, respectively, in the OC and OA conditions. Similarly, the orange and black lines show the averaged spectra of the noEEG channel, respectively, in the OC and OA conditions. The green vertical line aims at highlighting the similitude of the spectras and the equivalent IAF value.

The first aim of the study was to demonstrate the possibility of estimating cerebral parameters (i.e., IAF) by electrical activity recored from non-cerebral sites. Figure 5 reports the averaged spectra of the mean OC and OA conditions estimated from the Oz (EEG) and Neck4 (noEEG) channel. In particular, the red and blue lines show the averaged spectra of the EEG channel, respectively, in the OC and OA conditions. Similarly, the orange and black lines show the averaged spectra of the noEEG channel, respectively, in the OC and OA conditions. The green vertical line aims at highlighting the similitude of the EEG and noEEG spectras among the considered conditions (i.e., OC and OA), and how the Alpha peak decreases from OC (red and orange plots) to OA (blue and black plots). In particular, the averaged IAF estimated from the Oz (IAF_EEG_) and Neck4 channels (IAF_noEEG_) was the same, that is IAF = 11 Hz.

**Figure 6:**
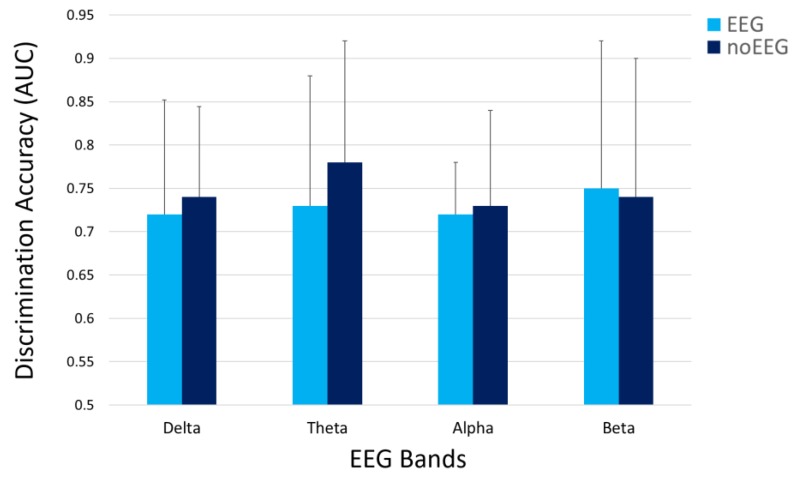
The averaged AUCs assumed values higher than 0.7 as a demonstration of good OA-OC discrimination in all the considered EEG bands. In addition, the t-tests on the averaged AUCs did not report significant (all *p* > 0.05) differences between the OC-OA discriminations obtained when using only the *EEG* or only the *noEEG* features.

In order to quantify the capability of non-cerebral electrical activity in assessing the participants’ states, thus discriminating between the OC and OA conditions, the averaged AUC_EEG_ and AUC_noEEG_ were calculated for the considered EEG bands. All of them assumed values higher than 0.7 (Table 3) as a demonstration of good conditions discrimination [34] when using only EEG, or only noEEG features. In addition, two-tail paired t-tests were performed on the averaged AUCs to assess eventual differences in terms of discrimination accuracy (i.e., AUC values) between EEG and noEEG features. No significant (all *p* > 0.05) differences were found between the OA-OC discriminations obtained when using only the EEG or only the noEEG features (Figure 6). 

**Figure 7:**
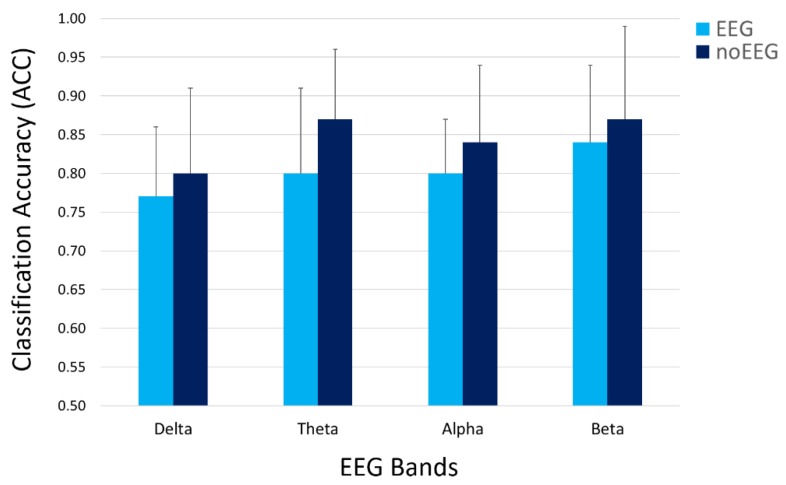
The averaged classification accuracies (ACCs) assumed values higher than 0.8 as a demonstration of high OA-OC classification in all the considered EEG bands. In addition, the t-tests on the averaged ACCs did not report significant (all *p* > 0.05) differences between the OC-OA classification obtained when using only the *EEG* or only the *noEEG* features.

To further assess the capability of the EEG and noEEG features in differentiating and classifying the participant’s states, we calculated the avereged classification accuracy (ACC) for the considered EEG bands. Considering that ACC = 0.5 means random classification and that almost all the averaged ACCs assumed values higher than 0.8 (Table 4). The results demonstrated how both the EEG and noEEG features were capable of high classification of the OA-OC conditions [32]. In addition, two-tail paired t-tests were performed on the averaged ACCs to assess eventual differences in terms of classification accuracy between the EEG and noEEG features. No significant (all *p* > 0.05) differences were found between the OA-OC classifications obtained when using the EEG and noEEG features (Figure 7).

## 4. Discussion

From an electrical point of view, human tissues are good conductive means, and the electrolytes are responsible for their conductivity. As a result, electrical potentials measured on different body locations, for example on the brain or on the neck, may show correlation and similar properties. In this regard, the proposed study aimed to address two issues. Firstly, we wanted to investigate the correlation between cerebral (EEG) and non-cerebral (noEEG) electrical activity, and the similarity in estimating the IAF parameter from the EEG or noEEG sites. Secondly, when significant (*p* < 0.05) correlations and similarities existed, the corresponding EEG and noEEG couples were used as features to create the mathematical models, respectively with only EEG or noEEG features, with the intent to compare and find out the capability of the two models in assessing the participants’ states (i.e OC or OA). The Pearson’s correlation and similiraty analysis showed the existence of noEEG locations in which the estimations of the IAF were exactly the same of those calculated from the corresponding EEG sites (green box in Figure 3). These aspects were also highlighted by the averaged spectra between the mean OA and mean OC conditions (Figure 4 and Figure 5). The EEG-noEEG couples in which the estimations of the IAF were identical, and were then used as an ensemble of features for the SWLDA classifier in order to validate their capability in discriminating the OC-OA conditions. In particular, the SWLDA was forced to shape the mathematical model by using only such features in order to assess the capability of the considered EEG and noEEG features in discrimintaing and classifying the users’ status. The two SWLDA models, one consisting of only EEG features, and the other of only noEEG features, were then employed to discriminate and classify the OC and OA experimental conditions. Both the cerebral (EEG) and non-cerebral (noEEG) features exhibited high discrimination (all AUCs > 0.7, Figure 6) and classification accuracy (all ACCs > 0.8, Figure 7) in the considered EEG bands (Delta, Theta, Alpha, and Beta), as demonstration of how they were able to significanly assess the users’ states. Furthermore, the t-tests between the averaged AUCs (Figure 6) and ACCs (Figure 7) did not report significant differences (all *p* > 0.05) in using either only EEG and only noEEG features for the OA-OC assessment. In other words, there was no difference in discriminating and classifying the OA-OC conditions using cerebral (EEG) or non-cerebral (noEEG) features in all the considered EEG bands. The capability of using non-cerebral locations would have an important impact on applications in everyday life. In fact, sensors and systems for EEG recordings are expensive and need specialized personnel. Additionally, the employment of non-cerebral sensors will make the user’s assessment less invasive and simpler. For example, user’s mental workload might be measured by using only a couple of electrodes on the neck rather than placing several sensors on the head and using an appropriate EEG cap in order to ensure the right pressure, comfort, and signal quality. Finally, the results demonstrated how appropriate study and selection of non-cerebral locations overcame the limitations of most of the current commercial locations (not designed for research or clinical purpose).

## 5. Conclusions

The proposed study demonstrated the existence of correlations between cerebral and non-cerebral electrical activity, and the possibility to estimate cerebral parameters (i.e., Individual Alpha Frequency—IAF) from non-cerebral sites thanks to the property of the electrical activity to propagate across the body through human tissues. Additionally, the study proved how some *noEEG* locations can be used, alternatively, to the *EEG* ones, to assess the participant’s states and overcome limitations of most of the current commercial (not designed for research or clinical purpose) EEG sensors and devices. The advantages of this result are very important and practical, especially for applications in real settings. In fact, the user’s mental states assessment would result in simpler, cheaper, and a less invasive result. Therefore, the next steps of this study will be the investigations of non-cerebral locations, including other body parts, for the user’s mental states measure under more realistic conditions, for example, while driving a car under different road and traffic situations, or stress conditions.

## Figures and Tables

**Table 1:** EEG bands definition by means of the individual alpha frequency (IAF) value.

EEG Band	f1	f2
Delta	0	IAF − 6
Theta	IAF − 6	IAF − 2
Alpha	IAF − 2	IAF + 2
Beta	IAF + 2	IAF + 16
Gamma	IAF + 16	IAF + 25

**Table 2:** The number of training and testing features for each frequency band and channel type.

Channel Type	Delta	Theta	Alpha	Beta
EEG	72	144	144	360
noEEG	64	128	128	320

**Table 3:** Summary of statistical analysis on the discrimination accuracy. Specifically, for each EEG band and features type, the area under the curve (AUCs) and the p-value was reported.

Features Type	Delta (*p* = 0.72)	Theta (*p* = 0.47)	Alpha (*p* = 0.88)	Beta (*p* = 0.93)
EEG	0.72	0.73	0.72	0.75
noEEG	0.74	0.78	0.73	0.74

**Table 4:** Summary of statistical analysis on the classification accuracy. Specifically, for each EEG band and features type, the ACCs and *p*-value were reported.

Features Type	Delta (*p* = 0.53)	Theta (*p* = 0.16)	Alpha (*p* = 0.42)	Beta (*p* = 0.55)
EEG	0.77	0.80	0.80	0.84
noEEG	0.80	0.87	0.84	0.87
